# The long non-coding RNA H19 induces hypoxia/reoxygenation injury by up-regulating autophagy in the hepatoma carcinoma cells

**DOI:** 10.1186/s40659-019-0239-2

**Published:** 2019-06-13

**Authors:** Chao Cui, Zhiyu Li, Dequan Wu

**Affiliations:** Department of General Surgery, The Second Affiliated Hospital of Harbin Medical University, Baojian Road, Harbin, 150086 China

**Keywords:** Long non-coding RNA H19, Hypoxia/reoxygenation, Autophagy, Hepatoma carcinoma cells

## Abstract

**Background:**

Long non-coding RNA H19 (H19) plays an important role by regulating protein expression in different tissues and organs of the body. However, whether H19 induces hypoxia/reoxygenation (h/R) injury via increase of autophagy in the hepatoma carcinoma cells is unknown.

**Results:**

H19 was expressed in the hepatoma carcinoma cells (Hep G2 and HCCLM3 cells) and its expression was most in 8 h/24R. The knockdown of H19 and 3-MA (an autophagy inhibitor) protected against h/R-induced apoptosis, cell damage, the expression of cleaved caspase-3 and cleaved caspase-9, the release of cytochrome *c* (Cyt *c*). The knockdown of H19 and 3-MA also decreased the autophagic vesicles (AVs) and the expression of Beclin-1 and the ration of LC3-II/LC3-I, and increased cell viability, the expression of Bcl-2 and P62 and the phosphorylation of PI3K, Akt and mTOR. In addition, chloroquine (CQ, an inhibitor of autophagy flux) markedly decreased formation of autophagy flux (the ration of LC3-II/LC3-I). The results of the knockdown of H19 group were similar to those of the 3-MA (or CQ) group. Rapamycin (a mTOR inhibitor, an autophagy activator) further down-regulated h/R-induced decrease of the phosphorylated PI3K, Akt and mTOR. The knockdown of H19 cancelled the effect of rapamycin. The overexpression of H19 further expanded h/R-induced increase of the ration of LC3-II/LC3-I and decrease of the phosphorylated PI3K, Akt and mTOR.

**Conclusions:**

Our results suggest that the long non-coding RNA H19 induces h/R injury by up-regulation of autophagy via activation of PI3K–Akt–mTOR pathway in the hepatoma carcinoma cells.

## Background

Ischemia/reperfusion (I/R) injury is a major cause of morbidity and mortality in many diseases such as stroke, myocardial infarction and acute renal tubular necrosis. Ischemic conditions result in ATP depletion and accumulation of toxic metabolites. Reperfusion causes production of reactive oxygen intermediates [1]. These alterations contribute to I/R injury, which is characterized by the presence of necrotic and apoptotic areas in the affected organs [1, 2]. Apoptosis has been recognized as a cellular mechanism of I/R injury [1, 2]. I/R can damage hepatocyte and decrease liver function [3], but I/R-induced damage of the hepatoma carcinoma cells plays a beneficial role in recovery of liver function.

Long non-coding RNAs (lncRNAs) belong to a newly discovered class of genes in the human genome that have been proposed to be key regulators of biological processes [4, 5]. LncRNAs are ncRNAs > 200 nucleotides long and do not encode proteins. Although only a handful of lncRNAs have been fully characterized, it is clear that they are involved in diverse biological processes [6]. Recent studies revealed that lncRNAs participate in the regulation of protein expression through functioning as the molecular decoys, the mediators of signaling pathways, the molecular guides for transcriptional co-activators and the scaffold for the formation of functional complex [7, 8]. Long non-coding RNA H19 (H19) expresses mainly in embryo [8, 9]. The abnormal expression of H19 has been found in several kinds of tumors such as gastric cancer [8, 10], liver cancer [8, 11], bladder cancer [8, 11] and choriocarcinoma [12].

Autophagy is a key factor in keeping the balance between synthesis, degradation, and recycling of cellular components [13, 14]. It has been suggested that excessive autophagy activation by apoptosis and cell death can induce the pathogenesis of diverse human diseases, for example liver disease, cancer, neurodegeneration disease, cardiovascular disease and aging [15–17].

It was reported that H19 induced cerebral I/R injury via activation of autophagy [8]. However, whether H19 plays a critical role in inhibiting proliferation and promoting apoptosis of the hepatoma carcinoma cells by up-regulating autophagy is unknown. This study investigated the effect of H19 on I/R and its possible mechanisms, including the autophagy and autophagy relative signaling pathway (PI3K–Akt–mTOR), in the hepatoma carcinoma cells.

## Materials and methods

### Drugs and reagents

3-Methyladenine (3-MA, an autophagy inhibitor) chloroquine (CQ, an inhibitor of autophagy flux) and rapamycin (Rapa, a mTOR inhibitor, an autophagy activator) were purchased from Sigma Chemical Co. (St. Louis, MO, USA). The antibodies of anti-pro-survival kinases phosphatidylinositol-3-OH kinase (PI3K), protein kinase B (Akt) and mammalian target of rapamycin (mTOR) were obtained from Cell Signaling Technology (Danvers, USA). The anti-Beclin-1, p62, cleaved caspase-3 and -9, Bcl-2, cytochrome *c* (Cyt *c*), microtubule associated protein light chain 3II and 3I (LC3II and LC3I) and β-actin were from Proteintech Group, Inc (Wuhan, China). H19 siRNA and control siRNA were from Santa Cruz (Bergheimer, Germany). Assay kits for aspartate aminotransferase (AST), alanine transaminase (ALT) and lactate dehydrogenase (LDH) were purchased from Nanjing Jiancheng Bioengineering Institute (Nanjing, China). Cell Counting Kit-8 (CCK-8) and Hoechst 33342 staining were obtained from Boster Bio-engineering Limited Company (Wuhan, China). Monodansylcadaverine kit (MDC) was purchased from keyGEN BioTECH (Nanjing, China). All other chemicals were from Sigma or Santa Cruz.

### Culture of the hepatoma carcinoma cells

The Hep G2 and HCCLM3 cells were purchased from the Cell Bank of the Chinese Academy of Sciences. Cells were cultured in growth medium DMEM containing 10% fetal bovine serum (FBS), 100 U/ml penicillin, and 100 mg/ml streptomycin. The experiments were performed when the cells reached 70–80% confluence between passages 6 and 10.

### Established the hepatoma carcinoma cells model of h/R

A hypoxic condition was produced by D-Hank solution (in mM: 5.37 KCl, 0.44 KH_2_PO_4_, 136.89 NaCl, 4.166 NaHCO_3_, 0.338 Na_2_HPO_4_, 5 d-glucose, pH 7.3–7.4 at 37 °C) saturated with 95% N_2_ and 5% CO_2_. The pH was regulated to 6.8 with lactate to mimic ischemic solution. The Hep G2 cells were put into a hypoxic incubator that was equilibrated with 1% O_2_/5% CO_2_/94% N_2_ for 4 h or 8 h or 12 h. After hypoxia, the culture medium in the Hep G2 cells was rapidly replaced with fresh DMEM with 10% fetal bovine serum (normoxic culture solution) for initiating reoxygenation for 24 h [18].

### Experimental protocols

The Hep G2 cells were randomly divided into the following four groups. Each group included eight samples (n = 8): (1) control group (control): the Hep G2 cells were cultured for 32 h with 10% fetal bovine serum-DMEM; (2) hypoxia/reoxygenation group (8 h/24R): the Hep G2 cells were exposed to hypoxic culture medium for 8 h and reoxygenated for 24 h by replacing the hypoxic culture medium with fresh DMEM with 10% fetal bovine serum; (3) 8 h/24R + H19 siRNA (or H19 siRNA control) group: H19 siRNA and corresponding control siRNA were transfected to the Hep G2 cells. The cells were then treated as those of group 2; (4) 8 h/24R + 3-MA group: the procedure was similar to that for group 2, except that 5 mM 3-MA were added in 24 h reoxygenation.

### Cell viability assay

Cell viability was measured by Cell Counting Kit-8 (CCK-8). Cells were seeded in 96-well plates at a concentration of 3 × 10^3^ cells/well. After 24 h of each treatment, 10 μl was added to each well of CCK-8 immediately. Subsequently, they were incubated for 2 h at 37 °C. Using a microplate spectrophotometer, the plates were read at 570 nm (A570) to determine their optical density.

### Analysis of cells in G0/G1 phase

In the present study, cell cycle progression was determined using propidium iodide methodology by flow cytometry. The cells were harvested with 0.25 g/l trypsin in 6-well plates, re-suspended in 10 ml PBS, 1 ml 70% ethanol was added, and the cells were then centrifuged (2000 rpm, 5 min, 4 °C) and washed with cold PBS. The cells were subsequently dyed with 100 μl RNAse (1 mg/ml) and then 400 μl PI (20 μg/ml). Following incubation for 30 min at room temperature, the samples were run on a 7 Laser SORP BD LSR II system (BD Biosciences, Franklin Lakes, NJ, USA) and the data was analyzed using CellQuest software (BD Biosciences).

### Measurement of AST, ALT and LDH activities

At the end of reoxygenation, cell culture medium was stored at − 80 °C until use. The activity of AST, ALT and LDH in the cell culture medium were measured spectrophotometrically with a commercially available assay kit (Nanjing Jiancheng Bioengineering Institute, Nanjing, China). All assays were conducted according to the manufacturer’s instructions [18].

### Apoptotic rate of cells by flow cytometry assay and Hoechst 33342 staining

The apoptotic rate was measured by flow cytometry as described previously [18, 19]. Cells were washed three times with ice-cold PBS, and then stained with annexin V-fluorescein isothiocyanate for 15 min at room temperature in 200 μl binding buffer. Next, 300 μl binding buffer was added, and the cells were stained with propidium iodide for 30 min at 4 °C. The fluorescence of the cells was analyzed by flow cytometry. The percentage of apoptotic cells was determined using Mod Fit LT software (Verity Software House Inc., Topsham, ME, USA).

Cells were analyzed for apoptosis after visualization of nuclei morphology with fluorescent DNA-binding dye Hoechst 33342, as described previously [19]. After treatment, cells were rinsed with PBS and incubated with 5 µg/ml Hoechst 33342 for 10 min. Nuclei were visualized at 400× magnification using fluorescent microscopy at an excitation wavelength of 330–380 nm. Apoptotic nuclei of cells were assessed by counting the number of cells that displayed nuclear morphology changes, such as chromatin condensation and fragmentation.

### MDC staining

Autophagic vacuoles were detected with MDC as described previously [20]. Cells were incubated with MDC (50 μM) in PBS at 37 °C for 20 min. After incubation, the cells were repeatedly washed (three times) with PBS and immediately analyzed by fluorescence microscope (excitation wavelength, 380 nm; emission filter, 525 nm).

### Western blotting analysis

The related protein expressions was measured by Western blot as described previously [13, 18, 19]. Briefly, equal amounts of proteins were subjected to sodium dodecyl sulfatepolyacrylamide gel electrophoresis and blotted on polyvinylidene fluoride membranes. The membranes were incubated with antibodies against cleaved-3 and -9, Bcl-2, Beclin-1, LC3II, p62, ATG 5, p-PI3K/t-PI3K, p-Akt/t-Akt, p-mTOR/t-mTOR and β-actin. The secondary antibody was goat anti-rat immunoglobulin G. The intensities of the protein bands were quantified by a Bio-Rad ChemiDoc™ EQ densitometer and Bio-Rad Quantity One software (Bio-Rad Laboratories). The protein concentration was quantified using the BCA Protein Assay kit (Beyotime, Nantong, China).

### Detection of Cyt *c* release from mitochondrial

Western blot analysis of Cyt *c* in the cytosolic fraction was performed as described previously [13, 18, 19]. Briefly, cells were harvested, washed twice with ice-cold PBS, and incubated in ice-cold Tris-sucrose buffer (0.35 mM sucrose, 10 mM Tris–HCl at pH 7.5, 1 mM EDTA, 0.5 mM dithiothreitol, 0.1 mM phenylmethylsulphonyl fluoride). After a 40 min incubation, cells were centrifuged at 1000×*g* for 5 min at 4 °C and the supernatant was further centrifuged at 40,000×*g* for 30 min at 4 °C. The supernatant was retained as the cytosolic fraction and analyzed by Western blot with a primary rat anti-Cyt *c* monoclonal antibody and a secondary goat anti-rat immunoglobulin G (Promage). β-actin expression was used as the control.

### Real-time PCR analysis

Total RNA was isolated using an RNeasy Mini Kit (Qiagen, Germantown, MD) and converted to cDNA with an iScriptTM cDNA Synthesis Kit (Bio-Rad, Hercules, CA). Real-time PCR was performed in an iCycler iQ5 apparatus (Bio-Rad) associated with the iCycler optical system software (version 3.1) using SYBR Green PCR Master Mix. The primers of H19 were 5′-TACAACCACTGCACTACCTG-3′ (forward) and 5′-TGGAATGCTTGAAGGCTGCT-3′ (reverse). The primers for β-actin were 5′-TCAGGTCATCACTATCGGCAAT-3′ (forward) and 5′-AAAGAAAGGGTGTAAAACGCA-3′ (reverse). The cycling conditions were: one cycle of 94 °C for 2 min; 30 cycles of 94 °C for 30 s, 60 °C for 40 s and 72 °C for 1 min; and 72 °C for 4 min. Relative mRNA quantification was calculated by using the arithmetic formula “2^−ΔΔCT^”, where ΔCT is the difference between the threshold cycle of a given target cDNA and an endogenous reference β-actin cDNA [18].

### Short interfering RNA (siRNA) transfection

Transfection of the Hep G2 cells by siRNA (H19 siRNA and corresponding control siRNA) was achieved by using the Lipofectamine™ 3000 transfection agent from Invitrogen (Burlington, ON). In brief, Hep G2 cells were seeded at equal number of cells (2.0 × 10^5^ per plate) in 60 mm^2^ plates with the medium containing 10% FBS. The cells were plated to form 60–70% confluent monolayers for siRNA transfection. siRNA and the transfection reagent complex were added to the serum-free medium for 4 h, and the transfection continued for another 24 h in serum-containing regular medium. After that, the cells were collected for detection of mRNA levels with real-time PCR analysis [21].

### Overexpression of H19

The H19 overexpression sequence si-H19 and the control si-NC were purchased from GenePharma (Shanghai GenePharma Co., Ltd., Shanghai, China). The transfection reagent Lipofectamine™ 3000 and interference sequence were mixed and added to the Hep G2 cells with the serum-free medium for 4 h, and the transfection continued for another 24 h in serum-containing regular medium. After that, the cells were collected for detection of mRNA levels with teal-time PCR analysis.

### Statistical analysis

All data were expressed as the mean ± SE and represented at least three independent experiments. Statistical comparisons were made using student’s *t*-test or one-way ANOVA followed by a post hoc analysis (Tukey test) where applicable. Significance level was set at p < 0.05.

## Results and discussion

**Fig. 1:**
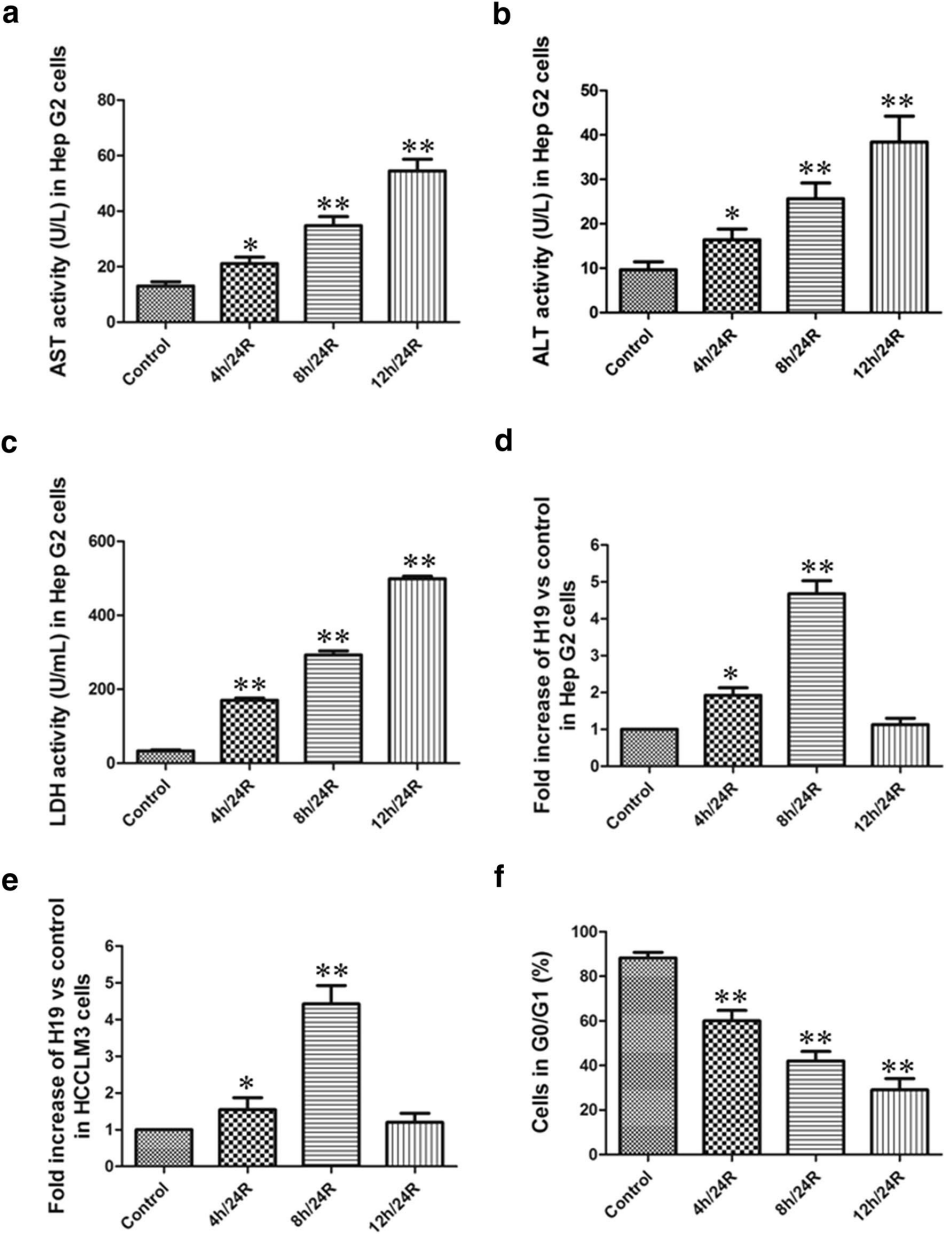
The change of AST, ALT and LDH activities in the Hep G2 cells and the level of H19 mRNA in the Hep G2 and HCCLM3 cells. AST (**a**), ALT (**b**) and LDH (**c**) activities were detected in the cell culture fluid. Data are mean ± S.E.M. of eight determinations. The level of H19 mRNA in the Hep G2 (**d**) and HCCLM3 cells (**e**). The level of H19 mRNA was observed by real-time PCR analysis. The Ct of H19 from different groups in the Hep G2 cells: control: 22.50; 4 h/24R: 22.00; 8 h/24R: 19.00; 12 h/24R: 21.50. The Ct of H19 from different groups in the HCCLM3 cells: control: 22.00; 4 h/24R: 21.50; 8 h/24R: 19.50; 12 h/24R: 21.50. The Hep G2 cells in G0/G1 phase (**f**). The cells in G0/G1 phase were detected using propidium iodide methodology. The data were from four independent experiments. *p < 0.05 vs. control group; **p < 0.01 vs. control group

Hypoxia/reoxygenation (h/R) injury can damage cell membrane and lead to the leakage of enzymes [22]. In the present study, we observed that during h/R, the activity of AST, ALT and LDH were significantly increased in culture medium of the Hep G2 cells. In addition, along with the prolongation of hypoxia time, the leakage of enzyme was also increased in the Hep G2 cells. This indicates the successful establishment of h/R model in the hepatoma carcinoma cells (Fig. 1a–c). We also observed that the level of H19 mRNA in the Hep G2 and HCCLM3 cells was markedly increased during h/R. And the level of H19 mRNA was the highest in the 8 h/24R group (Fig. 1d, e). Our data showed that the cells in G0/G1 phase were decreased during h/R (Fig. 1f). And the cells in G0/G1 phase were the lowest in the 12 h/24R group (Fig. 1f). These results suggested that H19 significantly promotes h/R injury in the hepatoma carcinoma cells and we chose 8 h/24R to establish h/R injury model in the following experiments.

Hepatic h/R causes hepatocytes necrosis and apoptosis [22]. The mitochondrial pathway is an important apoptotic pathway [18, 19, 23–25]. Cyt *c* is the initiating factor of mitochondrial apoptosis pathway. The Cyt *c* is released from injured mitochondria and triggers cytosolic caspase-3 activation through formation of the cytochrome *c*/Apaf-1/caspase-9-containing complex apoptosome and then lead to apoptosis [18, 19, 23–25]. Bcl-2 belong to a potent inhibitor of apoptosis and inhibit the mitochondria disruption and the subsequent Cyt *c* release, and the activation of caspase [18, 19, 23–25].

Recent studies have suggested the critical role of lncRNAs in the regulation of gene expression, which are shown to play an important role in the pathogenesis of tumors [4, 5, 8]. In another study, it was shown that lncRNAs, especially H19, promoted I/R injury [8]. There are several indications that lncRNAs may function as pro-apoptotic or anti-apoptotic regulators [5, 26].

**Fig. 2:**
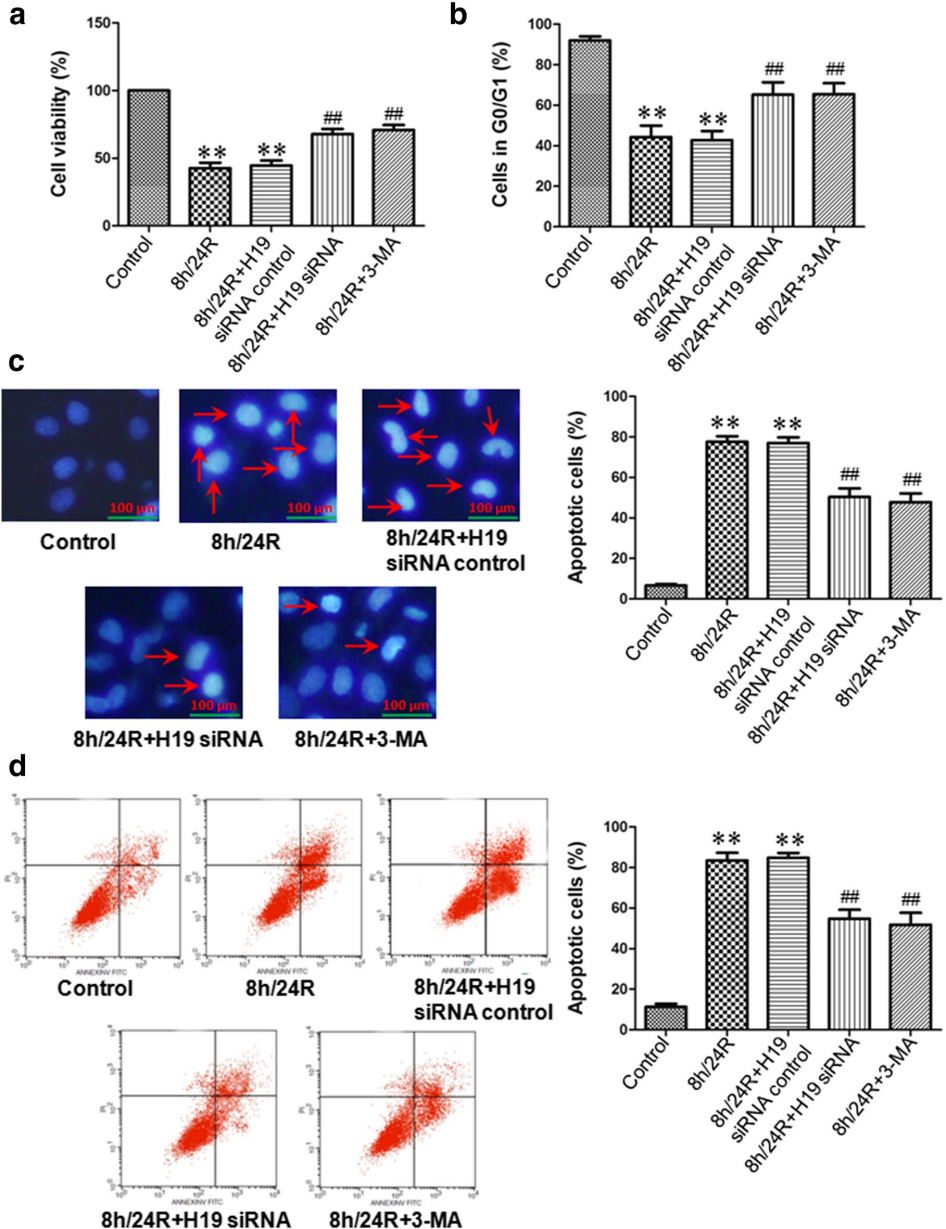
Knockdown of H19 increases cell viability and decreases apoptotic rate in the Hep G2 cells. H19 siRNA was used as the knockdown of H19, 5 mM 3-MA in 24 h reoxygenation was used to inhibit autophagy. **a** Knockdown of H19 increases cell viability. Cell viability was measured by CCK-8 kit. The cells incubated with control medium were considered 100% viable. All data are mean ± S.E.M. of eight determinations. **b** Knockdown of H19 increases the cells in G0/G1 phase. The cells in G0/G1 phase were detected using propidium iodide methodology. **c** Detection of nuclear morphology in apoptotic cells by Hoechst 33342 staining. Apoptotic cells were identified as cells with condensed, disrupted nuclei (arrow, Hoechst staining, ×400). Scale bar 100 μm. Apoptotic cells in at least five random fields were counted. **d** Apoptosis analyzed by flow cytometry. All data were from four independent experiments. **p < 0.01 vs. control group; ^##^p < 0.01 vs. 8 h/24R group

**Fig. 3:**
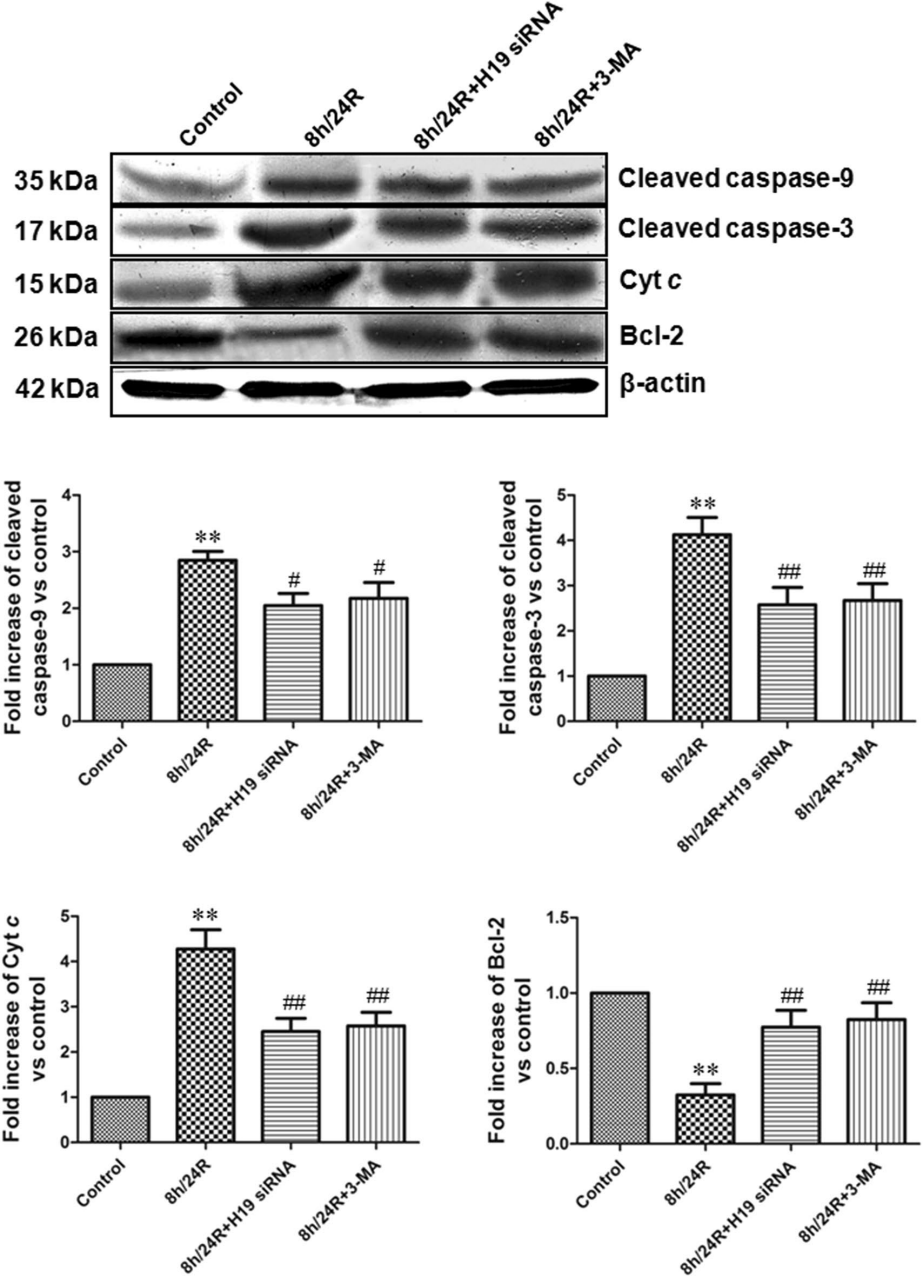
Knockdown of H19 inhibits the expression of pro-apoptotic factors and promotes anti-apoptotic factor in the Hep G2 cells. H19 siRNA was used as the knockdown of H19, 5 mM 3-MA in 24 h reoxygenation was used to inhibit autophagy. The expression of pro-apoptotic factors (cleaved caspase-3, -9 and Cyt *c*) and anti-apoptotic factor (Bcl-2) by western blot analysis. The intensity of each band was quantified by densitometry, and data were normalized to the β-actin signal. The expression levels in the control group were considered the basal levels, and the others are expressed as fold change from the control group. All data were from four independent experiments. **p < 0.01 vs. control group; ^#^p < 0.05 vs. 8 h/24R group; ^##^p < 0.01 vs. 8 h/24R group

Our results showed that the 8 h/24R decreased cell viability, the cells in G0/G1 phase and the expression of Bcl-2, increased the apoptotic rate and cleaved caspase-9, cleaved caspase-3 and Cyt *c* expressions in the Hep G2 cells. The 8 h/24R + H19 siRNA inhibited the 8 h/R-induced decrease of cell viability, the cells in G0/G1 phase and the expression of Bcl-2 and increase of the apoptotic rate and cleaved caspase-9, cleaved caspase-3 and Cyt *c* expressions in the Hep G2 cells (Figs. 2, 3). These results indicate that H19 promotes h/R injury-induced apoptosis by activation of the mitochondrial apoptotic pathway in the hepatoma carcinoma cells.

**Fig. 4:**
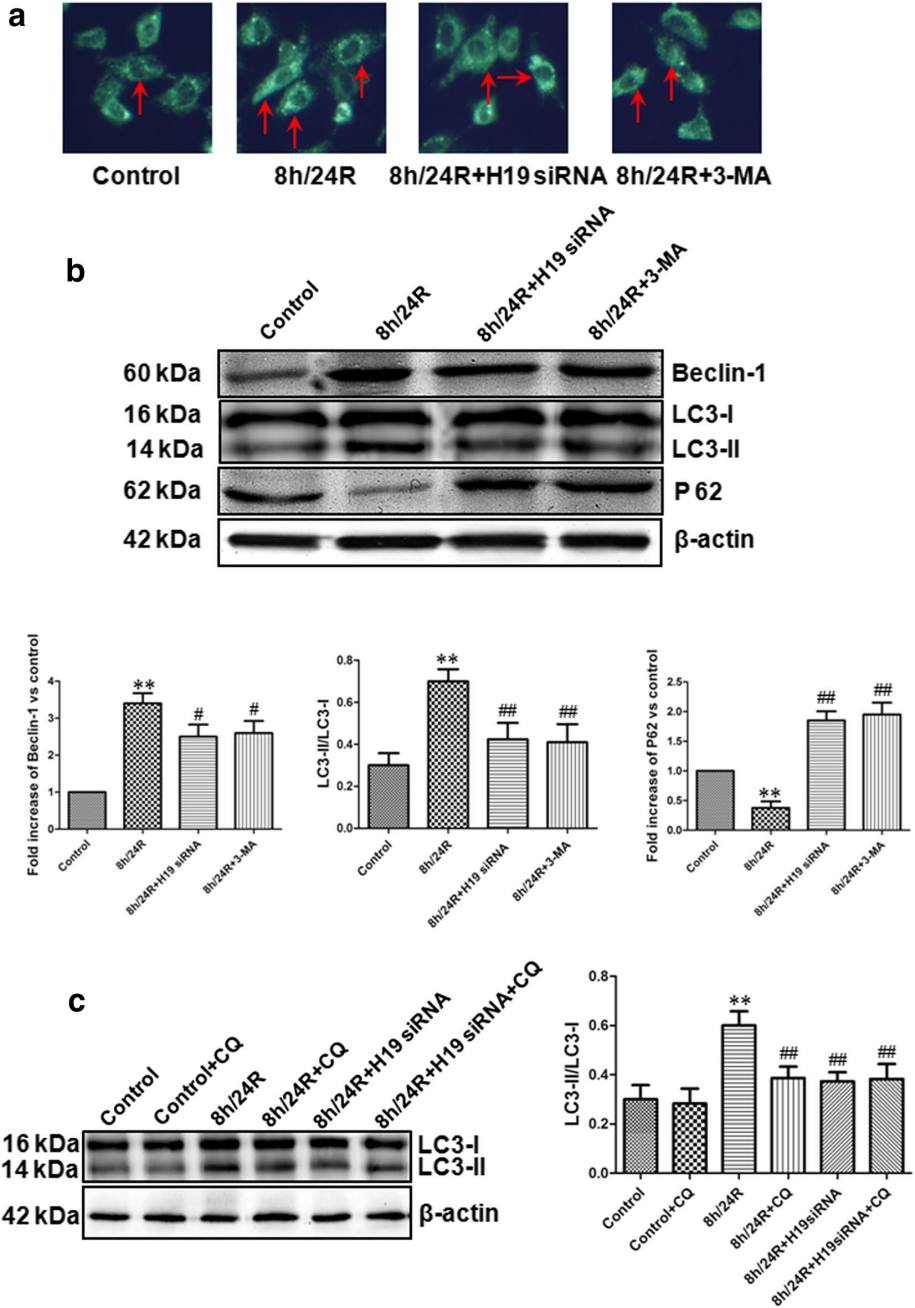
Knockdown of H19 decreases autophagy in the Hep G2 cells. H19 siRNA was used as the knockdown of H19, 5 mM 3-MA in 24 h reoxygenation was used to inhibit autophagy. **a** Acidic vesicular organelles were observed by MDC staining. Fluorescent dots indicate late autophagic vacuoles. The red arrows indicate the autophagic vacuoles (400× magnification). Scale bar = 10 μm. **b** The expression of autophagy related protein (Beclin-1 and p62) by western blot analysis. The intensity of each band was quantified by densitometry, and data were normalized to the β-actin signal. The expression levels in the control group were considered the basal levels, and the others are expressed as fold change from the control group. The ration of LC3-II/LC3-I was analyzed through western blot. **c** The autophagy flux (the ration of LC3-II/LC3-I) was detected by western blot analysis. 25 μM chloroquine (CQ) was used to inhibit an inhibitor of autophagy flux. All data were from four independent experiments. **p < 0.01 vs. control group; ^#^p < 0.05 vs. 8 h/24R group; ^##^p < 0.01 vs. 8 h/24R group

H/R (or I/R) increases autophagy. Excessive autophagy activation promotes cell apoptosis and death. Autophagy is characterized by the sequestration processes of cytoplasmic material within an autophagosome for degradation by lysosomes [27]. Autophagy acts as a pro-survival mechanism for maintaining normal cellular functions and serves as an adaptive response during various stress conditions, such as amino acid starvation, an unfolded protein response or viral infection [27]. Autophagosome formation requires two ubiquitin-like conjugation systems: the Atg 8 (microtubule-associated protein-1 light chain 3 [LC3]) conjugation system, and the Atg 5–Atg 12 conjugation system [28]. LC3 is as a marker for the detection of autophagosomes [29, 30]. Endogenous LC3 is present in two forms, LC3-I and LC3-II. LC3-I in turn is modified to a membrane bound form LC3-II to prompt its localization to autophagosomes [29, 30]. The increase of LC3-II shows the up-regulation of autophagy [29]. In contrast, the decrease of LC3-II shows the down-regulation of autophagy [29]. Beclin 1 also is as a mammalian autophagic gene and plays an important role in the autophagosome formation [29, 31]. Recent evidence suggests that the accumulation of p62 represents a convenient in vivo marker for impaired autophagy [32]. In this study, in the Hep G2 cells, we found that compared with the control group, AVs, the expression of Beclin-1 and the ration of LC3-II/LC3-I were significantly increased, while the expression of p62 was obviously decreased in the 8 h/24R group. Meanwhile, we also found that compared with the 8 h/24R, the 8 h/24R + H19 siRNA and 8 h/24R + 3-MA markedly inhibited formation of the AVs, decreased the expression of Beclin-1 and the ration of LC3-II/LC3-I and increased p62 expressions. The effect of the 8 h/24R + H19 siRNA on the autophagy relative index was similar to the 8 h/24R + 3-MA (Fig. 4a, b). Our data also showed that 8 h/24R + CQ (CQ, chloroquine, an inhibitor of autophagy flux) markedly decreased 8 h/24R-induced the ration of LC3-II/LC3-I and the effect of the 8 h/24R + H19 siRNA and the 8 h/24R + H19 siRNA + CQ on the the ration of LC3-II/LC3-I was similar to the 8 h/24R + CQ (Fig. 4c). In addition, the overexpression of H19 further expanded h/R-induced increase of the ration of LC3-II/LC3-I (Fig. 6b). These data suggest that H19 increased h/R injury and apoptosis by up-regulating autophagy in the hepatoma cells.

**Fig. 5:**
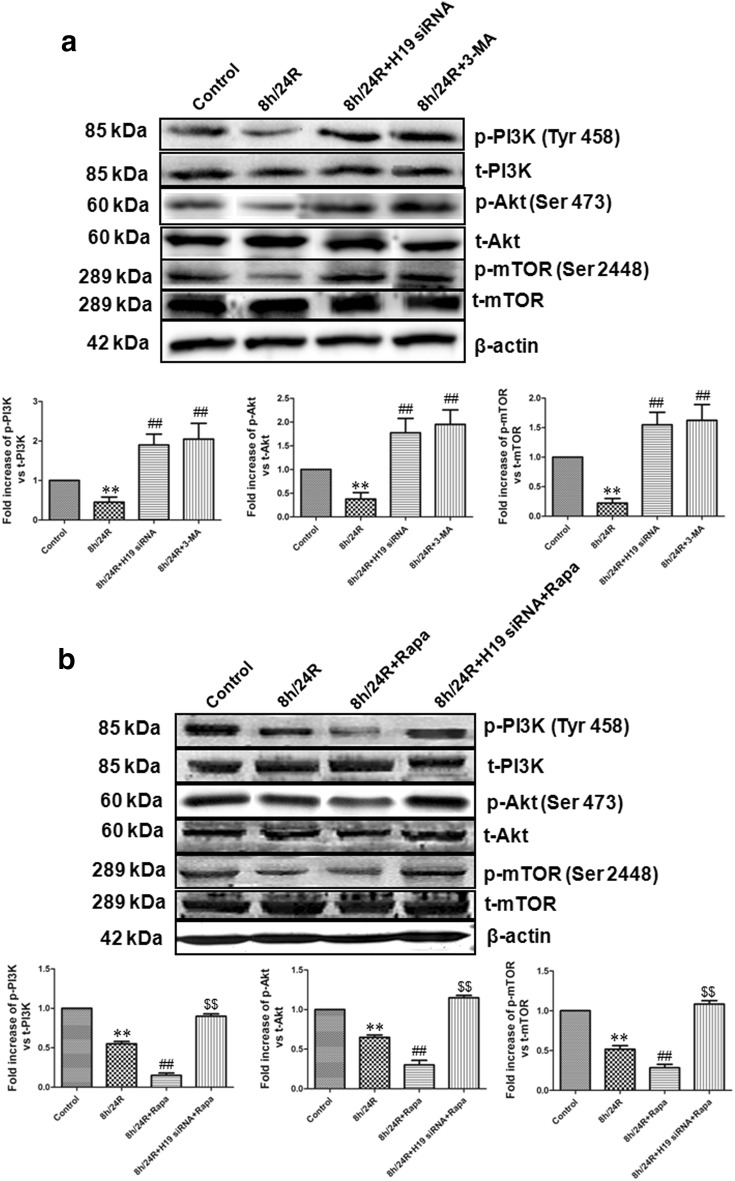
Knockdown of H19 up-regulates PI3K–Akt–mTOR pathway in the Hep G2 cells. H19 siRNA was used as the knockdown of H19, 5 mM 3-MA in 24 h reoxygenation was used to inhibit autophagy and 50 nM Rapa (rapamycin) in 24 h reoxygenation was used to inhibit mTOR and activate autophagy. **a** Knockdown of H19 increases the phosphorylation of PI3K, Akt and mTOR was detected using western blot analysis. The graphs represent the optical density of the bands of phospho-PI3K (p-PI3K), Akt (p-Akt) and mTOR (p-mTOR) normalized with the expression of total-PI3K (t-PI3K), Akt (t-Akt) and mTOR (t-mTOR). The phosphorylated levels in the control group were considered the basal levels, and the others are expressed as fold change from the control group. All data were from four independent experiments. **p < 0.01 vs. control group; ^##^p < 0.01 vs. 8 h/24R group. **b** Knockdown of H19 cancelled the effect of Rapa on the phosphorylation of PI3K, Akt and mTOR. The graphs represent the optical density of the bands of phospho-PI3K (p-PI3K), Akt (p-Akt) and mTOR (p-mTOR) normalized with the expression of total-PI3K (t-PI3K), Akt (t-Akt) and mTOR (t-mTOR). The phosphorylated levels in the control group were considered the basal levels, and the others are expressed as fold change from the control group. All data were from four independent experiments. **p < 0.01 vs. control group; ^##^p < 0.01 vs. 8 h/24R group; ^$$^p < 0.01 vs. 8 h/24R + Rapa group

**Fig. 6:**
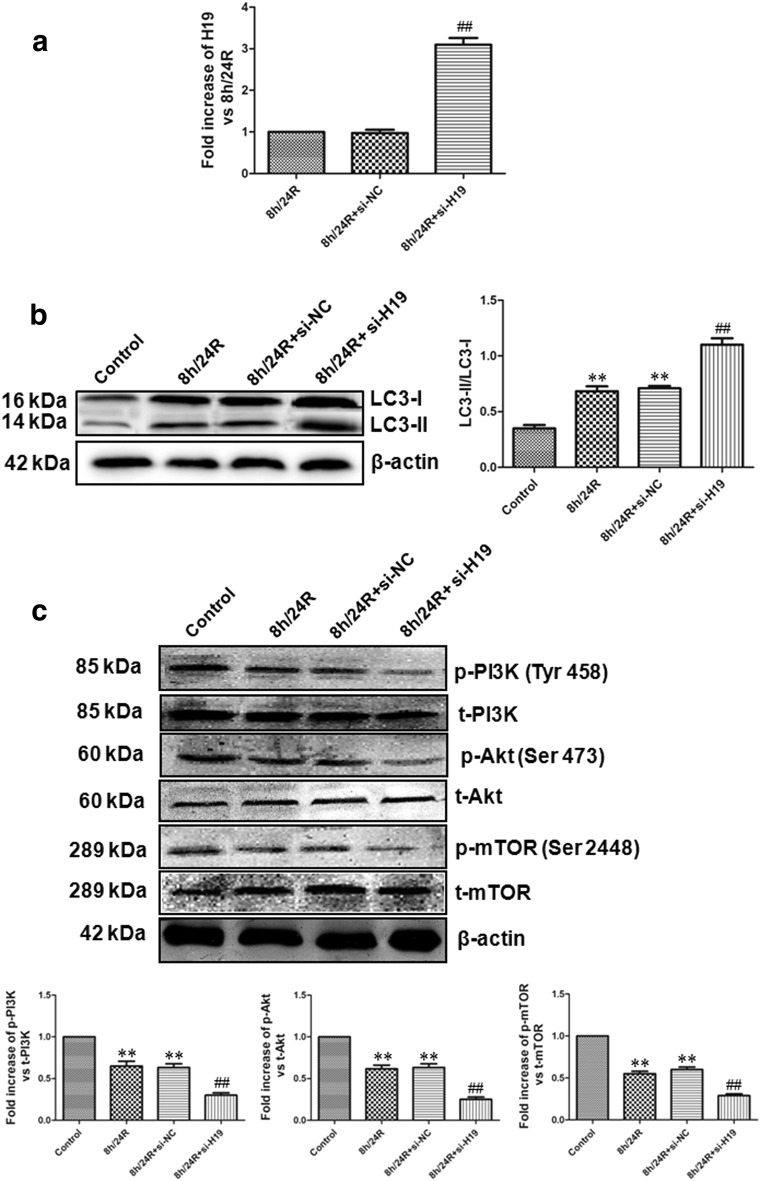
Overexpression of H19 increases the ration of LC3-II/LC3-I and down-regulates PI3K–Akt–mTOR pathway in the Hep G2 cells. Si-H19 was used as the overexpression of H19. **a** The overexpression of H19 was observed by real-time PCR analysis. All data were from four independent experiments. ^##^p < 0.01 vs. 8 h/24R group or 8 h/24R + si-NC group. **b** The ration of LC3-II/LC3-I was detected by western blot analysis. The intensity of each band was quantified by densitometry, and data were normalized to the β-actin signal. **c** The phosphorylation of PI3K, Akt and mTOR was detected using western blot analysis. The graphs represent the optical density of the bands of phospho-PI3K (p-PI3K), Akt (p-Akt) and mTOR (p-mTOR) normalized with the expression of total-PI3K (t-PI3K), Akt (t-Akt) and mTOR (t-mTOR). The phosphorylated levels in the control group were considered the basal levels, and the others are expressed as fold change from the control group. All data were from four independent experiments. **p < 0.01 vs. control group; ^##^p < 0.01 vs. 8 h/24R group

To further indicate H19 induced h/R injury and apoptosis by increase of autophagy in the hepatoma carcinoma cells, we explored relevant signaling pathways of autophagy. mTOR, a protein kinase, regulates cell growth and metabolism and also is the best characterized regulator of autophagy [14, 30]. mTOR is an up-stream negative regulator of autophagy [33]. The activation of the pro-survival kinases phosphatidylinositol-3-OH kinase (PI3K) and protein kinase B (Akt) increases mTOR activity, thereby down-regulating autophagy, indicating that the pathway for PI3K–Akt–mTOR is important for autophagy regulation [33]. Previous studies have also indicated that the activation of PI3K–Akt pathway up-regulates mTOR and decreases autophagy in different cell types [34, 35]. We showed here that in the Hep G2 cells, 8 h/24R decreased the phosphorylated activity of PI3K, Akt and mTOR (Fig. 5a). However, the 8 h/24R + H19 siRNA and 8 h/24R + 3-MA (an autophagy inhibitor) significantly increased the phosphorylated activity of PI3K, Akt and mTOR (Fig. 5a). The effect of the 8 h/24R + H19 siRNA on the phosphorylated activity of PI3K, Akt and mTOR was similar to the 8 h/24R + 3-MA (Fig. 5a). 8 h/24R + Rapa (rapamycin, a mTOR inhibitor, an autophagy activator) further expanded h/R-induced decrease of the phosphorylated PI3K, Akt and mTOR (Fig. 5b). The 8 h/24R + H19 siRNA + Rapa cancelled the effect of rapamycin (Fig. 5b). The overexpression of H19 further expanded h/R-induced decrease of the phosphorylated PI3K, Akt and mTOR (Fig. 6c). Taken together, these findings suggest that H19 plays an important role in the promotion of h/R injury and apoptosis by up-regulation of autophagy via the inhibiting PI3K–Akt–mTOR pathway in the hepatoma carcinoma cells.

## Conclusions

In conclusion, our present study demonstrates that long non-coding RNA H19 (H19) promotes h/R injury is associated with the up-regulation of autophagy through down-regulating PI3K–Akt–mTOR pathway in the hepatoma carcinoma cells. These findings provide new insight into the prevention and therapy of hepatic tumors.

## Data Availability

Please contact author for data and materials requests.

## Abbreviations

H19: long non-coding RNA H19
h/R: hypoxia/reoxygenation
CQ: chloroquine
Cyt *c*: cytochrome *c*
AVs: autophagic vesicles
I/R: ischaemia/reperfusion
lncRNAs: long non-coding RNAs
FBS: fetal bovine serum
CCK-8: Cell Counting Kit-8
siRNA: short interfering RNA
LC3: microtubule-associated protein-1 light chain 3
Rapa: rapamycin
